# Efficiency of Electronic Nose in Detecting the Microbial Spoilage of Fresh Sardines (*Sardinella longiceps*)

**DOI:** 10.3390/foods13030428

**Published:** 2024-01-29

**Authors:** Haitham S. Al-Hooti, Ismail M. Al-Bulushi, Zahir H. Al-Attabi, Mohammad S. Rahman, Lyutha K. Al-Subhi, Nasser A. Al-Habsi

**Affiliations:** 1 Department of Health Inspection, Muscat Municipality in Bosher, Muscat Municipality, P.O. Box 609, Seeb 111, Oman; haitham.s.alhooti@mm.gov.om; 2 Department of Food Science and Nutrition, College of Agricultural and Marine Sciences, Sultan Qaboos University, P.O. Box 34, Al-Khod 123, Oman; zaherr@squ.edu.om (Z.H.A.-A.); shafiur@squ.edu.om (M.S.R.); lyutha@squ.edu.om (L.K.A.-S.); habsin@squ.edu.om (N.A.A.-H.)

**Keywords:** sardines, microbial spoilage, bacterial count, electronic nose

## Abstract

The assessment of microbial spoilage in fresh fish is a major concern for the fish industry. This study aimed to evaluate the efficiency and reliability of an electronic nose (E-nose) to detect microbial spoilage of fresh sardines (*Sardinella longiceps*) by comparing its measurements with Total Bacterial Count (TBC), Hydrogen Sulfide (H_2_S) producing bacterial count and Trimethylamine Oxide (TMAO) reducing bacterial count after variable storage conditions. The samples were stored at 0 °C (0, 2, 4, 6, and 8 days) and 25 °C (0, 3, 6, and 9 h), while day 0 was used as a control. The E-nose measurements were analyzed by Principal Component Analysis (PCA), Linear Discriminant Analysis (LDA) and Artificial Neural Network (ANN). Microbial counts increased significantly and simultaneously with the changes in E-nose measurements during storage. The LDA and ANN showed a good classification of E-nose data for different storage times at two storage temperatures (0 °C and 25 °C) compared to PCA. It is expected as PCA is based on linear relationships between the factors, while ANN is based on non-linear relationships. Correlation coefficients between E-nose and TBC, TMAO-reducing bacterial and H_2_S-producing bacterial counts at 0 °C were 0.919, 0.960 and 0.915, respectively, whereas at 25 °C, the correlation coefficients were 0.859, 0.945 and 0.849, respectively. These positive correlations qualify the E-nose as an efficient and reliable device for detecting microbial spoilage of fish during storage.

## 1. Introduction

Microbial spoilage is one of the main fish quality defects in post rigor mortem. The formation of Volatile Organic Compounds (VOCs), such as dimethyl sulfide, 2,3-pentadione, hexanal, and 2-nonanol, causes the rejection of fish due to their unpleasant odors [1]. Different VOC indicators or markers have been identified in different fish samples [2,3]. Therefore, VOCs are used as an indicator for fish quality assessment (i.e., freshness and spoilage).

Sardine is found in a wide variety of habitats throughout the world. It contains high levels of Polyunsaturated Fatty Acids (PUFAs), such as Omega-3. The distinct flavor and odor of sardine are due to the presence of PUFAs [4]. In addition, alcohol (i.e., 1-penten-3-ol, 1-methoxy-2-propanol, trans-2-penten-1-ol, and 3-pentanol) may contribute to the distinctive odor of Pilchard (i.e., a sardine species found primarily in the Mediterranean Sea) [5]. Additionally, dimethyl sulfide and methyl disulfide were identified as prominent VOCs in fresh sardine and serve as the primary components of the marine/iodized odor in raw sardine. Fresh sardines were reported to have different classes of VOCs, for example, aldehydes, alcohols, ketones, hydrocarbons, and sulfur-containing compounds [1,5]. The odor of spoiled sardines was described as fishy [4], when a 2-ethylfuran compound could be a possible cause [5].

Microbial spoilage can be assessed by different methods, such as enumeration of Total Bacterial Count (TBC) [6], H_2_S-producing bacterial count, and Trimethylamine Oxide (TMAO)-reducing bacterial count [7]. The TBC was considered a reference and an index for fish spoilage [8]. Moreover, the H_2_S-producing bacterial count was related to fish spoilage causing off-odor problems [9,10]. Hydrogen sulfide, methyl mercaptan, methyl sulfide, and dimethyl sulfide can cause putrid odors [2]. Furthermore, reduction of TMAO bacteria (i.e., TMAO to TMA) was considered an excellent indicator of fish spoilage, and these were responsible for fishy odor [4,10,11,12,13]. However, these methods are labor-, time- and resource-intensive. Thus, there is a need to develop non-destructive, fast, effective and simple techniques to assess the quality of fresh fish.

The electronic nose (E-nose) is an instrument that comprises an array of electronic chemical sensors with a specificity for odors. It has an appropriate pattern-recognition system capable of recognizing simple or complex odors [14]. The E-nose draws the volatile compounds from a headspace, and then these are passed through the sensors for odor detection. The signals from the sensors are then transferred to a computer, and then the data are analyzed using mathematical algorithms such as Principal Component Analysis (PCA), Linear Discriminant Analysis (LDA), Partial Least Squares (PLS), Support Vector Machines (SVMs), Cluster Analysis (CA), Functional Discriminant Analysis (FDA), and Artificial Neural Networks (ANNs) [15]. The human olfactory system can detect odors at certain limits or thresholds; however, the E-nose can detect odors beyond those limits. E-nose was widely used in monitoring air quality, the quality of treated wastewater, food freshness and spoilage detection, and disease detection in plants and humans [16].

In fish, E-nose was used to differentiate anchovy quality [17], fish freshness [18,19,20,21] and microbial spoilage [22]. The E-nose data were compared with the standard microbial analyses, such as total bacterial count, mesophilic bacteria, and Total Volatile Basic Nitrogen (TVB-N) and Acid Value (AV) [18]. However, none of the previous studies evaluated the fresh fish spoilage (i.e., sardines) with their spoilage indicators, such as TMAO and H_2_S bacterial counts and their correlations with the E-nose measurements. It could be concluded from the previous studies that the E-nose showed capability to evaluate the quality of fish, and it could have a potential capability to detect quality deterioration due to microbial spoilage. Therefore, this study aimed to evaluate the efficiency and reliability of E-nose to detect microbial spoilage of fresh sardines (*Sardinella longiceps*) when stored at 0 °C or 25 °C. The capability was determined considering VOC formation due to the growth of TBC, H_2_S-producing bacteria, and TMAO-reducing bacteria.

## 2. Materials and Methods

### 2.1. Fish Samples

Fresh sardines (*Sardinella longiceps*) were purchased from local fish markets in Muscat, Oman, within 12 h of landing. Fish were placed in ice, and they were transported to the laboratory within 40 min, and the height and weight of the fish were measured immediately. Sardines were about 18 to 23 ± 2 cm in length and 80 to 120 ± 5 g in weight. The fish were then placed in airtight plastic containers and stored at 0 °C for 8 days and at 25 °C for 9 days. Samples were analyzed at 2-day and 3-hour intervals during storage at 0 °C and 25 °C, respectively. All analyses were ceased by the appearance of offensive spoilage odor. Six lots of sardines (i.e., three lots for storage studies at 0 °C and three lots for storage studies at 25 °C) weighing 6 kg per treatment were used. Sardine samples were bought at different times while maintaining the same sources and the same methods of catching and handling.

### 2.2. Microbial Analyses

To enumerate TBC, the whole fish was cut into cube-size pieces, mixed well, and randomly, 25 g was mixed with 225 mL of maximum recovery diluent (Oxoid, Hampshire, UK). After serial dilutions, 0.1 mL of the sample suspension was spread on tryptone soya agar (Oxoid, Hampshire, UK) supplemented with 2% NaCl (Oxoid, Hampshire, UK), and plates were incubated at 25 ± 1 °C for 72 ± 2 h [23]. To enumerate H_2_S-producing bacteria, a fish sample was prepared following the same protocol as that for the total bacterial count. After a serial dilution, 0.1 mL of the sample suspension was spread on iron agar consisting of 2% bacteriological peptone (Oxoid, Hampshire, UK), 0.3% Lab-lemco powder (Oxoid, Hampshire, UK), 0.3% yeast extract (Oxoid, Hampshire, UK), 0.03% ferric citrate (BDH chemicals, Poole, UK), 0.04% L-cysteine (Fisher Scientific, Loughborough, UK), 0.05% sodium chloride bacteriological grade (Oxoid, Hampshire, UK) and 1.5% bacteriological agar (Oxoid, Hampshire, UK) [24]. Plates were incubated at 25 ± 1 °C for 72 ± 2 h. Black colonies were considered to be real H_2_S-producing bacteria [24].

Trimethyl amine oxide-reducing bacteria were enumerated on TMAO medium consisting of 2% bacteriological peptone (Oxoid, Hampshire, UK), 0.3% Lab-Lemco powder (Oxoid, Hampshire, UK), 0.3% yeast extract (Oxoid, Hampshire, UK), 0.4% sodium chloride bacteriological grade (Oxoid, Hampshire, UK), 0.4% potassium dihydrogen orthophosphate (BDH chemicals, Poole, UK), 0.6% di-potassium hydrogen orthophosphate trihydrate (BDH chemicals, Poole, UK), 0.05% magnesium sulfate anhydrous (Sigma, Kobe, Japan), 0.5% trimethylamine N-oxide dihydrate (Sigma, Kobe, Japan), and 1.5% bacteriological agar (Oxoid, Hampshire, UK) [24,25]. The protocol was the same as that used for TBC. The plates were incubated at 25 ± 1 °C for 72 ± 2 h, and the yellow colonies were considered as TMAO-reducing bacteria [24].

### 2.3. Electronic Nose Measurements

A Cyranose 320^™^ (Cyrano Science, Inc., Pasadena, CA, USA) with 32 carbon black polymer sensors was used to determine VOCs as produced by bacterial growth. The whole sardines were placed in an airtight plastic container with a 0.2-inch-diameter hole in the center of the lid (this hole was sealed and opened for headspace gas sampling). The E-nose needle was inserted through the hole to withdraw the volatile compounds created in the headspace. The E-nose parameters were optimized as per the procedure of Rahman [26]: baseline purge, 25 s; sample draw, 30 s; first sample gas purge, 50 s; second sample gas purge, 40 s, and second air intake purge, 10 s. A syringe filter with a diameter of 25 mm and pore size of 0.45 µm (Membrane Solutions, Auburn, WA, USA) was used between the E-nose needle and snout to trap any moisture. Drift of the sensors was corrected every day considering the room air as reference. All sensors were selected, and the data were further analyzed.

#### 2.3.1. Principal Component Analysis and Linear Discriminant Analysis

The E-nose data were analyzed using PCA and LDA by setting the data set from E-nose signals of all 32 sensors. Sensor responses were taken in relation to the baseline of purge gas using Equation (1). Room air was used as a reference and purge gas. At each storage time, 3 fish were used, with 5 readings on each fish. The PCA was performed by utilizing all scores from 32 sensors. Moreover, Eigenvalues were used to explain the PC total variance and to decide the number of PCs. The number of PCs was selected by utilizing a scree plot (i.e., when there was a negligible decrease in Eigenvalue as PCs were increased). Similar producers were followed in the case of the LDA. The LDA is a supervised learning algorithm that showed better classification than PCA in E-nose. Sensor response was standardized using Equation (1):

(1) $$Sensor\;response=\frac{R_{max}-R_{o}}{R_{o}}$$

where *R_max_* is maximum resistance and *R_o_* is baseline purge resistance.

#### 2.3.2. Artificial Neural Network and Correlation of E-Nose and Microbial Counts

The data from E-nose signals were prepared for ANN analysis using Attribute-Relation extension. The data were attributed by recognizing classes of storage times. A multilayer perception model, which uses backpropagation to classify instances, was used to conduct ANN of E-nose data based on storage time classification. The network consisted of an input, a hidden layer and an output. The confusion matrix of instances and the correct classification were obtained. The linear regression prediction was conducted for the correlation between microbial counts and E-nose. The value of the correlation coefficient was used to test the efficiency of the E-nose, and the root mean square error (RMSE) (Equation (2)) showed how the data fit the regression line.

(2) $$RMSE=\sqrt{\sum_{i=1}^{n}\frac{{\left({ŷ}_{i}-Y_{i}\right)}^{2}}{n}}$$

where ${ŷ}_{i}$ is the measurement or sample value, $Y_{i}$ is the corresponding prediction value, and n is the sample size.

### 2.4. Statistical Analysis

The microbial data were expressed using the logarithm of colony forming unit per gram (CFU/g) and represented as mean and standard deviation. Significant differences (*p* < 0.05) between means were tested using ANOVA one-way, least significant difference, ANOVA one-way and Duncan multiple range test (DMRT) using SPSS ver.18 (IBM, New York, NY, USA). The PCA and LDA were conducted using PAST ver.4.10 [27]. The ANN models and non-linear correlation were performed by WEKA ver.3.9 [28].

## 3. Results and Discussion

### 3.1. Microbial Analyses

#### 3.1.1. Changes in Bacterial Counts at 0 °C

**Figure 1:**
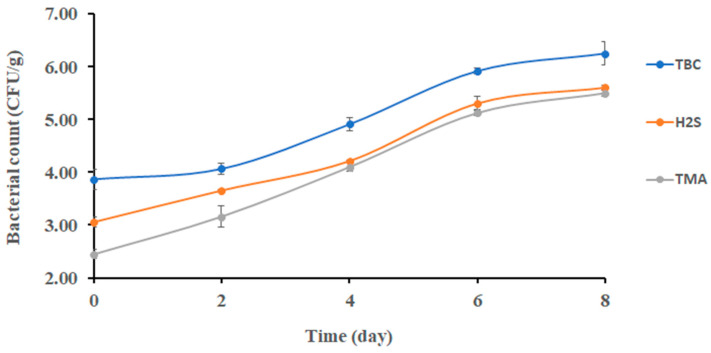
Changes in bacterial counts in sardines stored at 0 °C.

Figure 1 shows the changes in the TBC of sardines stored at 0 °C. The initial bacterial count of sardines at 0 days was 3.90 ± 0.20 log CFU/g; then, it significantly increased (*p* < 0.05) to 5.91 ± 0.07 log CFU/g after 6 days of storage. At the end of storage, the TBC reached 6.25 ± 0.22 log CFU/g. This finding partially agreed with Campos [29], who found that psychrotrophic bacteria counted approximately 5.00 log CFU/g after 8 days of storage, and the initial bacterial count was approximately 2.70 log CFU/g. However, the result of this study was lower than the result of Erken and Özden [30], who observed that the total bacterial count reached 5.37 log CFU/g after 9 days of storage. The TBC of quality food should not exceed 5.00 log CFU/g [31]. Accordingly, sardine lost its good microbial quality after 4 days of storage at 0 °C.

The initial count of TMAO (i.e., reducing bacteria) was 2.45 ± 0.29 log CFU/g (Figure 1). It increased significantly (*p* < 0.05) to 4.10 log CFU/g from day 2 to day 4. Thereafter, no significant change was observed. The TMAO-reducing bacteria such as *Shewanella putrefaciens* and *Photobacterium phosphoreum* were limited at the initial storage stage of sardines, which could explain the gradual change in TMAO-reducing bacteria as observed in the current study [29]. Moreover, *Shewanella putrefaciens* was found to be a stronger spoiling bacterium with the ability to reduce TMAO in sardines (*Sardina pilchardus*) [7].

Changes in H_2_S-producing bacteria were observed after 6 days of storage, and it was a different trend compared with TMAO-reducing bacteria (i.e., 4 days) (Figure 1). This could be explained as bacterial spoilage being initiated by TMAO-reducing bacteria. This finding was in agreement with Ólafsdóttir [32], who found a noticeable change in H_2_S- producing bacterial growth in redfish (*Sebastes marinus*) after 5 days of storage. The initial and final counts of H_2_S-producing bacteria in sardines (*Sardina pilchardus*) were 4.0 and 4.9 log CFU/g, respectively [30], when stored on ice for 9 days. This was close to the counts of 3.06 and 5.60 log CFU/g, respectively, as observed in the current study. Moreover, H_2_S-producing bacteria were found to increase in gilt-head sea bream (*Sparus aurata*) stored at different temperatures (0, 8, 15 °C) [33].

#### 3.1.2. Changes in Bacterial Counts at 25 °C

**Figure 2:**
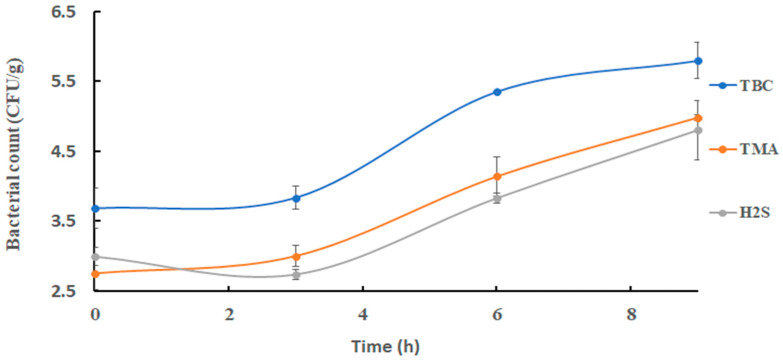
Changes in bacterial counts in sardines stored at 25 °C.

Changes in TBC in sardines at 25 °C are shown in Figure 2. The initial bacterial count of 3.69 ± 0.29 log CFU/g increased significantly (*p* < 0.05) to 5.79 ± 0.26 log CFU/g at the end of storage. This was close to the results of 4 log CFU/g as observed at 0 h during storage of Indian mackerel at the same storage temperature as used in the current study [34]. Thereafter, TBC was decreased to an acceptable quality of sardines after 6 h [31]. Trimethyl amine oxide-reducing bacterial count increased significantly (*p* < 0.05) from 2.76 ± 0.24 log CFU/g at 0 h to 4.14 ± 0.28 log CFU/g after 6 h, and it remained almost constant at 4.98 ± 0.05 log CFU/g at the end of storage (Figure 2). *Shewanella putrefaciens* was found to be the main TMAO-reducing bacterium in fish and responsible for fishy odors [7,29,35]. Hydrogen-sulfide-producing bacteria increased from an initial count of 3.00 ± 0.13 log CFU/g to 4.80 ± 0.43 log CFU/g at the end of storage (Figure 2).

### 3.2. Electronic Nose Analyses

#### 3.2.1. Principle Component Analysis

**Figure 3:**
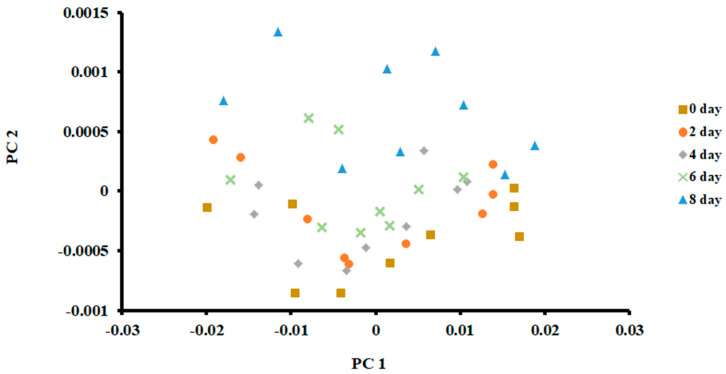
PCA plot of the first two principal components (PC) of the E-nose signals of sardines stored at 0 °C.

The PCA plot of the samples stored at 0 °C is shown in Figure 3. The first two principal components (PCs) were used to classify fish at different days of storage. It was based on the scree plot (i.e., Eigenvalue less than 1). The first PC explained 99.65% of total variances, while the second PC explained 0.22%.

As shown in Figure 3, it was difficult to separate fish when stored for different days. Only samples after 8 days of storage could be separated, although some data points overlapped with the 6 days of storage. The PCA is an unsupervised method and does not consider the non-linearity of the labelled data [36]. Chantarachoti et al. [20] also noticed that the PCA plot showed overlapping between days 1, 6 and 9 of storage in Alaska pink salmon when stored in slush ice. They did not explain why there was an overlap between these storage days. However, they pointed out that classification difficulty could be due to the complexity of the volatile compounds formed due to the spoilage of fish. However, data points were shifted upward with the increase in storage. This relative shift indicated that volatiles formed due to spoilage could be recognized by PCA, although a clear separation was difficult to observe. Jun [37] showed a similar shift when silver carp were stored in ice at 4 °C for 6.4 days. In contrast, a horizontal shift of the data points in the PCA plot was observed in the case of anchovies stored at 4 °C for 15 days [17].

**Figure 4:**
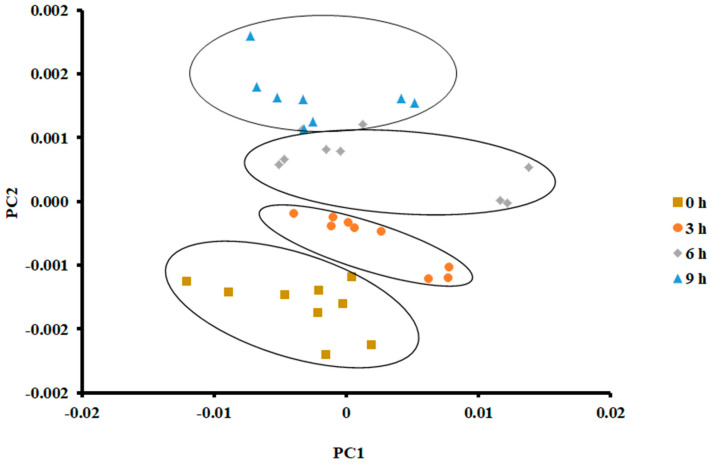
PCA plot of the first two principal components (PC) of the E-nose signals of sardines stored at 25 °C.

On the other hand, PCA showed a clear separation in the case of storage at 25 °C for 9 h (Figure 4). The vertical shift of clustered data (i.e., 4 clusters) was clear. PC 1 and PC 2 explained 95.83% and 3.092% of the variance, respectively. Tian [38] observed similar results when PCA was used to classify different hairtail fish when stored at 15 °C for different durations (i.e., first two storage days, 0.5 and 1 day). They grouped the stored fish after 2.5 and 3 days within the same class.

In the current study, there was a slide overlap between the data sets at the 6 h cluster and 9 h cluster. This could be attributed to the formation of similar volatile compounds with little change in their intensities. Chantarachoti [20] grouped three classes when Alaska pink salmon was stored at 14 °C for 0, 1, 2, and 3 days. They could not separate the classes of 0 and 1 days. Amari [17] grouped three classes when anchovies were stored at 4 °C within 15 days of storage. The first class included 1 and 3 days, the second class included 5 and 7 days, and the third class included 9–15 days.

An in-depth explanation of the overlap of the observed data set during different storage days could be explored by measuring volatiles by GC-MS [39]. El Barbri [19] explained the scattering and overlap of the data during storage time. They identified two effects: the formation of various types of volatiles during storage and the change in environmental temperature and humidity around the sample. In the current study, the temperature of the sensors was maintained at 40 °C, and the humidity of the overhead gas was filtered out of the gas sample before entering the E-nose. In addition, the temperature of the sample was controlled using an incubator instead of ice. It was interesting to see that PCA could classify samples stored at 25 °C compared to those stored at 0 °C. This could be due to the intensity of the volatiles formed during storage at a higher temperature.

#### 3.2.2. Linear Discriminant Analysis

**Figure 5:**
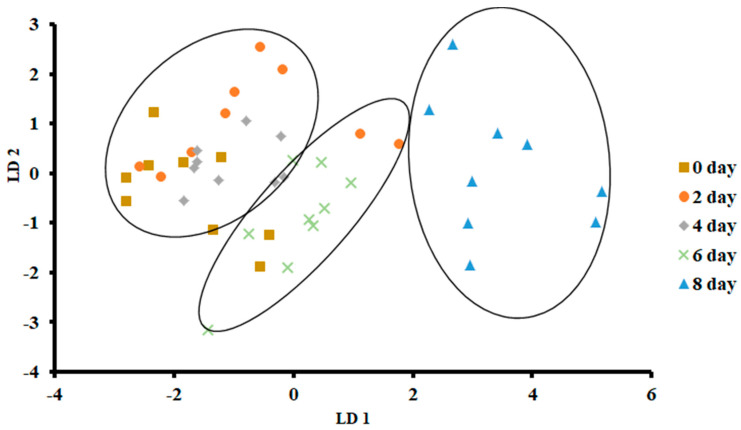
Biplot of linear discriminant analysis (LDA) of E-nose signals of sardines stored at 0 °C.

Figure 5 shows the plot of LDA at 0 °C. The variance in LD1 was 83.66%, while the variance in LD2 was 10.86%. In contrast to PCA, LDA separated three clusters, as the first one included days 0, 2 and 4, while the second cluster included day 6, and the third one included day 8. There was no overlap of the data set as observed for day 8, while in the other two classes, there was some overlap of the data set.

**Figure 6:**
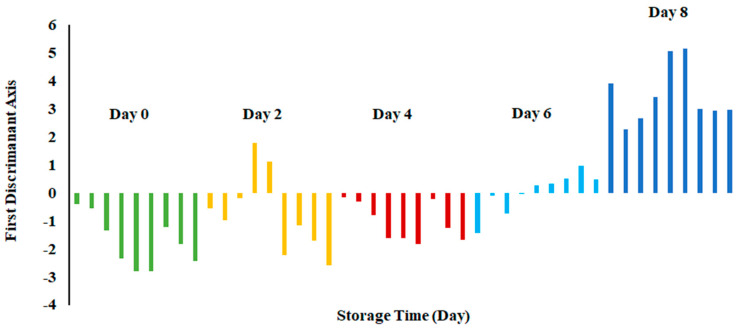
Bar plot of the first discriminant axis for sardines stored at 0 °C as function of time.

Figure 6 shows the bar plot of the first discriminant score as a function of storage at 0 °C. This shows a clear separation at day 8, while day 6 shows the transition. However, PCA showed overlap in all classes (Figure 3). The overlap could be due to similar volatiles with relatively similar intensities within the neighboring clusters. However, strong fishy odor was observed after day 6, as evidenced by the sensory perception.

Overall, LDA showed better classification than PCA. This could be due to the supervised training used in the case of LDA, although both considered linear models [40]. Similar findings were also reported by Zhu [36] in the case of freshness of Chinese mitten crab stored at 4 °C. Moreover, the greatest classification was obtained in freshness of anchovies when LDA was used as compared to PCA, although both methods gave a similar number of groups [17]. The LDA showed a horizontal shift from left (fresh) to right (spoiled).

**Figure 7:**
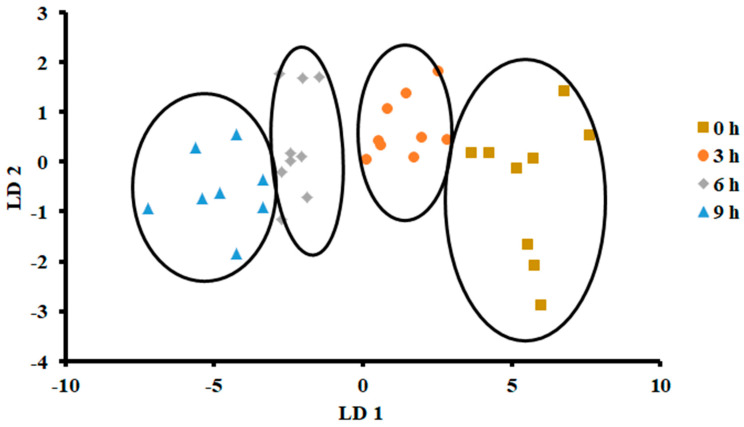
Biplot of linear discriminant analysis (LDA) of E-nose signals of sardines store at 25 °C.

**Figure 8:**
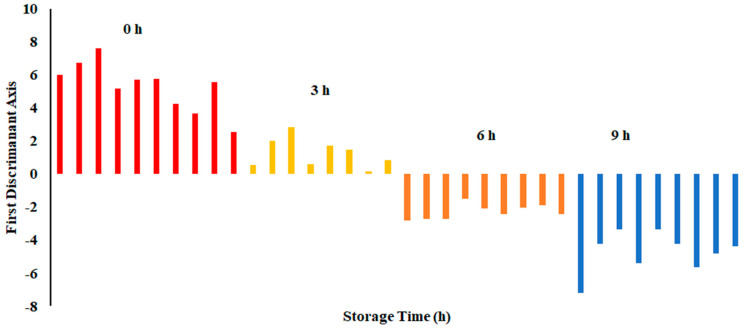
Bar plot of the first discriminant axis for sardines stored at 25 °C as function of time (hours).

The plot of LDA at 25 °C is shown in Figure 7. The variance in the first LD1 was 98.07%, while the second LD2 explained 1.849%. Similarly to PCA, LDA separated four classes considering hours stored (i.e., first class was 0 h, second class was 3 h, third class was 6 h, and fourth class was 9 h). The LDA did not show any overlap between the classes. Figure 8 shows the bar plot of the first discriminant score as a function of storage hours (i.e., for sardine stored at 25 °C). A clear separation between four classes was observed, and 6 and 9 h observed on the opposite side. Linear models, such as LDA and PCA, showed a good classification for fish stored at 25 °C. In the literature, limited studies showed the performance of LDA on fish when stored at ambient temperature (i.e., 25 °C).

#### 3.2.3. Artificial Neural Network

ANN was used to classify the E-nose signals, considering the high non-linearity of the data set. Table 1 shows the confusion matrix of the ANN classification for sardine stored at 0 °C. The ANN considered five classes as 0, 2, 4, 6 and 8 days, and it gave a 69% correct classification. In the case of day 0, two outliers (i.e., false values) were observed (Table 1). Similarly, day 2 and day 4 showed six outliers, while nine instances (i.e., true values) should be observed in each class. However, ANN classified nine correct instances (i.e., true values) on days 6 and 8 (i.e., 100% correct classification of instances). The total number of instances was 45. The low ability of classification in the initial storage period indicated the complexity of the volatiles, or it could be due to the low intensity of the volatiles in the early storage period. The low intensity of the volatiles could have been below the sensitivity of the E-nose sensors. However, the accuracy of the ANN classification was similar to that of the LDA classification. Hosseini [41] observed a 91% classification in the case of fish, while in this study, we observed only 69%. However, their storage temperature was 4 °C (i.e., refrigerator), which was higher than the storage temperature (i.e., 0 °C) used in this study. The increased storage temperature can provide better classification, as we have demonstrated earlier between 0 °C and 25 °C due to the high intensity of VOCs.

The confusion matrix of ANN at 25 °C is demonstrated in Table 2. The ANN gave a 100% correct classification. There were outliers or false values (i.e., total of 36 instances). It was emphasized that ANN is an effective method for freshness classification of fish using E-nose technology [41]. Among the different methods used to evaluate the accuracy of the E-nose classification of TVB-N content in freshwater fish freshness, ANN showed higher prediction accuracy [37].

### 3.3. Comparison between Electronic Nose and Microbial Growth

The correlation coefficient (R^2^) between E-nose signals (i.e., days or hours as a class) and microbial indicators can be seen in Table 3. It varied from 0.849 to 0.960. The RMSE varied from 0.319 to 0.525. It is clear that the R^2^ between E-nose and microbial group counts was higher with sardines stored at 0 °C compared to 25 °C in all cases of microbial counts. At 0 °C, the highest R^2^ was found between TMAO-reducing bacterial count and E-nose (e.g., 0.96), while at 25 °C, the highest correlation coefficient for sardine stored at 25 °C was between H_2_S-producing bacterial count and E-nose (e.g., 0.94). This discrepancy could be attributed to the volatiles produced by different bacterial groups at each temperature. For instance, TMA was found to be a more apparent volatile at low temperatures, while at high temperatures, H_2_S was more apparent [42].

Treberg [42] observed a high TMAO concentration in fish at low storage temperatures, while elevation of the storage temperature decreased TMAO concentration. The correlation coefficient between TBC and E-nose at 0 °C agreed, as observed by Han [18] (i.e., 0.914). At a higher temperature (e.g., 15 °C), Tian [38] found a high correlation coefficient of 0.91 between the E-nose signals and aerobic bacterial counts in the case of hair-tailed fish. In the previous studies, E-nose signals were highly correlated with the total bacterial count; nevertheless, earlier, there was no attempt to correlate E-nose signals with the H_2_S and TMA bacteria. It is, therefore, the novelty of this current study, and the current study needs to be extended to include more fish species and to determine the correlations between E-nose and other microbial spoilage indicators.

## 4. Conclusions

The microbial counts showed a significant increase in all microbial groups when samples were stored at 0 and 25 °C. TMAO-reducing bacteria showed an earlier increase when samples were stored at 0 °C. LDA and ANN analyses of E-nose data clearly showed distinct classes of storage time in the case of samples stored at 25 °C, while earlier storage period classification was difficult for the samples stored at 0 °C. In addition, LDA and ANN gave better classification than PCA. Higher positive correlations were found between E-nose and all counts of microbial groups at 0 °C than those at 25 °C. The current work found that E-nose could be a reliable technique to detect fresh fish microbial spoilage, as evident from the high correlations with the total and spoilage bacterial counts.

## Figures and Tables

**Table 1:** Confusion matrix of artificial neural network (ANN) classification of sardines stored at 0 °C.

** Classified as	a	b	c	d	e
a	7 *	2	0	0	0
b	3	3 *	2	1	0
c	0	3	3 *	3	0
d	0	0	0	9 *	0
e	0	0	0	0	9 *

* Correctly classified instances (i.e., true values) were 31, and the percentage was 68.8%, while incorrectly classified instances were 14, and the percentage was 31.11%. ** a (0 day), b (2 day), c (4 day), d (6 day), e (8 day) are storage days. Rows are actual values, and columns are predicted values.

**Table 2:** Confusion matrix of artificial neural network (ANN) classification of sardines stored at 25 °C.

** Classified as	a	b	c	d
a	9 *	0	0	0
b	0	9 *	0	0
c	0	0	9 *	0
d	0	0	0	9 *

* Correctly classified instances (i.e., true values) were 45, and percentage was 100%. ** a (0 h), b (3 h), c (6 h), d (9 h) are storage times. Rows are actual values, and columns are predicted values.

**Table 3:** Relationship between E-nose data and microbial indicators for sardines stored at 0 °C and 25 °C.

Storage Temperature	0 °C			25 °C		
Performance	TBC *	H_2_S **	TMA ***	TBC *	H_2_S **	TMA ***
R ^a^	0.919	0.915	0.960	0.859	0.945	0.849
RMSE ^b^	0.414	0.429	0.341	0.548	0.319	0.525

^a^ Correlation coefficient ^b^ root mean squared error * Total bacterial count ** H_2_S-producing bacterial count *** TMAO-reducing bacterial count. All correlations between microbial groups and E-nose for two storage temperatures were performed by utilizing whole training data set.

## Data Availability

Data is contained within the article.
